# Stereoselectivity of Aldose Reductase in the Reduction of Glutathionyl-Hydroxynonanal Adduct

**DOI:** 10.3390/antiox8100502

**Published:** 2019-10-22

**Authors:** Francesco Balestri, Vito Barracco, Giovanni Renzone, Tiziano Tuccinardi, Christian Silvio Pomelli, Mario Cappiello, Marco Lessi, Rossella Rotondo, Fabio Bellina, Andrea Scaloni, Umberto Mura, Antonella Del Corso, Roberta Moschini

**Affiliations:** 1 Biochemistry Unit, Department of Biology, University of Pisa, via S. Zeno 51, 56127 Pisa, Italy; francesco.balestri@unipi.it (F.B.); vito-00@hotmail.it (V.B.); mario.cappiello@unipi.it (M.C.); rossellarotondo@libero.it (R.R.); umberto.mura@unipi.it (U.M.); roberta.moschini@unipi.it (R.M.); 2 Interdepartmental Research Center Nutrafood “Nutraceuticals and Food for Health”, University of Pisa, 56124 Pisa, Italy; 3 Proteomics & Mass Spectrometry Laboratory, ISPAAM-CNR, Via Argine 1085, 80147 Napoli, Italy; giovanni.renzone@ispaam.cnr.it (G.R.); andrea.scaloni@ispaam.cnr.it (A.S.); 4 Department of Pharmacy, University of Pisa, via Bonanno 6, 56126 Pisa, Italy; tiziano.tuccinardi@unipi.it (T.T.); christian.pomelli@unipi.it (C.S.P.); 5 Department of Chemistry and Industrial Chemistry, University of Pisa, via G. Moruzzi, 13, 56124 Pisa, Italy; marco.lessi@unipi.it (M.L.); fabio.bellina@unipi.it (F.B.)

**Keywords:** aldose reductase, 4-hydroxy-2-nonenal, 3-glutathionyl-4-hydroxynonenal, inflammation

## Abstract

The formation of the adduct between the lipid peroxidation product 4-hydroxy-2-nonenal (HNE) and glutathione, which leads to the generation of 3-glutathionyl-4-hydroxynonane (GSHNE), is one of the main routes of HNE detoxification. The aldo-keto reductase AKR1B1 is involved in the reduction of the aldehydic group of both HNE and GSHNE. In the present study, the effect of chirality on the recognition by aldose reductase of HNE and GSHNE was evaluated. AKR1B1 discriminates very modestly between the two possible enantiomers of HNE as substrates. Conversely, a combined kinetic analysis of the glutathionyl adducts obtained starting from either 4R- or 4S-HNE and mass spectrometry analysis of GSHNE products obtained from racemic HNE revealed that AKR1B1 possesses a marked preference toward the 3S,4R-GSHNE diastereoisomer. Density functional theory and molecular modeling studies revealed that this diastereoisomer, besides having a higher tendency to be in an open aldehydic form (the one recognized by AKR1B1) in solution than other GSHNE diastereoisomers, is further stabilized in its open form by a specific interaction with the enzyme active site. The relevance of this stereospecificity to the final metabolic fate of GSHNE is discussed.

## 1. Introduction

Lipid peroxidation is one of the well-known toxic consequences of cell oxidative stress [1]. This process generates hydrophobic aldehydic compounds such as alkanals, alkenals and hydroxyalkenals that are able to induce cellular damage through the irreversible modification of proteins and nucleic acids [2]. Among lipid peroxidation products, 4-hydroxy-2-nonenal (HNE) is one of the most abundant; its ability to interact with and modify several proteins (from enzymes to transcription factors and cytoskeletal proteins) determines a variety of cellular responses [3]. Increased levels of HNE have been detected in several human diseases, including neurodegenerative, cardiovascular and metabolic disorders [4,5,6]. Indeed, protein carbonylation, occurring as a consequence of the generation of adducts with lysine, cysteine and histidine residues, has been recognized as one of the preeminent toxic actions of HNE [7]. The advance in detecting techniques, with immunohistochemistry as a relevant one, has given a strong impulse to the evaluation of the presence of HNE-protein adducts, and thus to the understanding of their role in the pathology of several diseases [8]. Indeed, recent evidence clearly indicates that the beneficial effects exerted by various antioxidants, both in vivo and in vitro, can derive from a decrease of HNE-protein adducts [9,10]. All these considerations suggest that the removal of HNE is relevant to human health [3].

The detoxification of HNE may occur through either redox transformation of its aldehydic group, or conjugation with thiol-containing compounds including glutathione (GSH) [11]. The catalytic action of aldehyde dehydrogenases, alcohol dehydrogenases and aldo-keto reductases has already been demonstrated to assist the redox transformation of the aldehydic group of HNE, with a selectivity that depends on cell type [12,13,14,15,16,17]. On the other hand, various glutathione S-transferase (GST) isoforms have been described as able to catalyze the generation of glutathionyl-HNE adduct (GSHNE) [18,19]. Reduction of the GSHNE aldehydic group has been reported to be catalyzed by the aldo-keto reductase aldose reductase (E.C.1.1.1.21; AKR1B1) [16], which generates a molecular species (glutahionyl-1,4-dihydroxynonane (GSDHN)) that is considered a trigger of the Nuclear Factor kappa-light-chain-enhancer of activated B cells (NF-κB) mediated inflammatory response [20,21]. This effect is the rationale for the anti-inflammatory activity associated with various AKR1B1 inhibitors in several cell systems [22]. Recently, the ability of the short-chain dehydrogenase carbonyl reductase 1 (CBR1) to catalyze both the reduction and the oxidation of GSHNE [23,24] has been also reported. In cardiovascular tissues, extensive GSHNE generation has been demonstrated to be the primary route of HNE removal [25,26]. Experimental evidence suggests that further reduction of GSHNE catalyzed by AKR1B1 elicits vascular smooth muscle cell (VSMC) growth [20,27]. An abnormal proliferation of VSMC is currently considered to be a factor contributing to several vascular disorders such as atherosclerosis, hypertension and vein graft disease [28,29].

**Figure 1:**
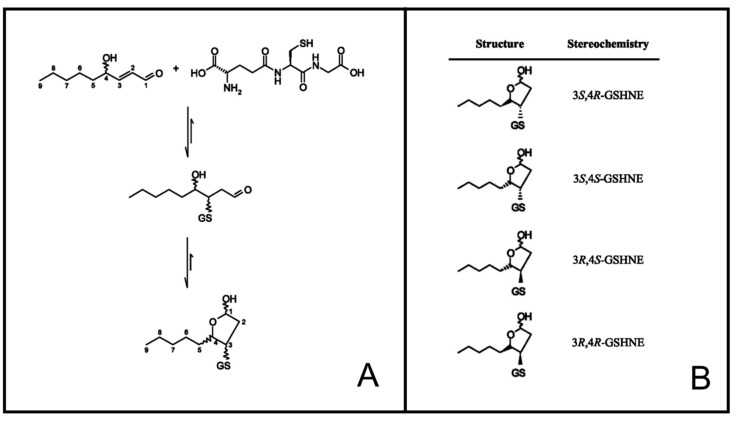
Reaction of 4-hydroxy-2-nonenal (HNE) with glutathione (GSH). (**A**) The reaction of GSH adduction on C3 of HNE followed by intramolecular cyclization to generate the corresponding hemiacetal species. (**B**) The structures of different 3-glutathionyl-4-hydroxynonane (GSHNE) diastereoisomers generated upon adduction.

Despite the potential for lipid peroxidation to generate both enantiomers of HNE (4*R* or 4*S*), the resultant chirality can highly affect the corresponding molecular metabolism and the compound ability to interact with other cellular components. In particular, a selective oxidation of 4*R*-HNE was reported to occur in brain mitochondria as a result of ALDH5 stereospecificity [30]. On the other hand, 4*S*-HNE has been indicated as the most efficient enantiomer in irreversibly inactivating glyceraldehyde-3-phosphate dehydrogenase [31]. Finally, a modest preference for 4*S*-HNE has been demonstrated in the generation of GSHNE when the reaction is catalyzed by the GST A4-4 isoform [32]. Worth mentioning is the fact that the reaction of HNE with GSH (Figure 1) generates a novel chiral center in GSHNE at the site of molecular adduction (C3 atom) [33], and various GST isoforms have been demonstrated to display differences in the stereoselectivity of GSHNE generation [32]. In particular, among the four possible anomeric GSHNE couples generated upon adduction of HNE to GSH (Figure 1), GSTA A4-4 has been shown to produce only the 3*S*-containing couples, while GST P1-1 and GST A1-1 have led to the generation of many possible products, although to different relative extents [32]. The abovementioned evidences highlight the contribution of chirality in HNE metabolism and in the subsequent biotransformations in GSHNE.

Concerning the specific relevance of chirality to the metabolism of GSHNE, we recently pointed out the ability of CBR1 to preferentially oxidize specific diastereoisomers among those generated upon the spontaneous adduction of HNE to glutathione [24]. 

Here we report evidence that AKR1B1 is also able to catalyze the reduction of the aldehydic group of GSHNE with a marked stereospecifity; a rationale for this preference is given using a molecular modeling approach.

## 2. Materials and Methods

### 2.1. Materials 

GSH, bovine serum albumin (BSA), D,L-glyceraldehyde (GAL), 2nd generation Grubbs catalysts, lipase acrylic resin from *Candida antarctica*, *R/S*-oct-1-en-3-ol, Met-Arg-Phe-Ala (MRFA) peptide, sequencing-grade trypsin and precoated silica gel polyethylene terephthalate (PET) foils were purchased from Sigma Aldrich (St. Louis, MO, USA). Bradford reagent was purchased from Bio-Rad (Hercules, CA, USA). YM10 membranes (10 kDa cut-off value) were purchased from Amicon Millipore (Darmstadt, Germany). All inorganic chemicals were of reagent grade from BDH (VWR International Ltd., Poole Dorset, UK). L-idose and NADPH were from Carbosynth (Compton, England). All solvents were of high performance liquid chromatography (HPLC)-grade from J.T. Baker Chemicals (Fisher Scientific, Thermo Fisher, Waltham, MA, USA). Anhydrous dichloromethane (DCM) was obtained from the commercial HPLC-grade DCM by distillation on CaH_2_.

### 2.2. Determination of AKR1B1 Activity 

Human recombinant AKR1B1 (*h*AKR1B1) activity was evaluated at 37 °C as previously described [34], measuring the absorbance at 340 nm, whose decrease with time is linked to NADPH oxidation The assay mixture assembled in 0.25 M sodium phosphate buffer (pH 6.8) contained 0.18 mM NADPH, 0.5 mM ethylenediamine tetra-acetic acid (EDTA), 0.42 M ammonium sulfate and 4.7 mM GAL as substrate. Measurements were performed through a Libra S60 spectrophotometer (Biochrom Ltd., Cambridge, England), adopting a differential extinction coefficient for NADPH oxidation at 340 nm of 6.22 mM^−1^ cm^−1^. One unit of enzyme activity is defined as the amount of enzyme that catalyzes a conversion of 1 µmol substrate/min under the above-described assay conditions. 

### 2.3. Expression and Purification of AKR1B1

*h*AKR1B1 was expressed, purified to electrophoretic homogeneity and stored as previously described [35]. Before use, the purified enzyme (specific activity 5.3 U/mg) was extensively dialyzed on YM10 membranes against a 10-mM sodium phosphate buffer (pH 7.0).

### 2.4. Synthesis of HNE Stereoisomers

#### 2.4.1. Analytical Methods

Gas Liquid Chromatography (GLC) analyses were performed using a Dani GC 1000 instrument (Dani Instruments S.p.A., Cologno Monzese, Italy) equipped with a programmed temperature vaporizer injector and recorded with a Dani DDS 1000 data station. The following capillary columns were used: (i) Agilent J&W HP-5ms column (30 m × 0.25 mm i.d. × 0.25 μm); (ii) Agilent J&W DB-5 column (30 m × 0.25 mm i.d. × 1 μm); (iii) Alltech AT-35 FSOT column (30 m × 0.25 mm i.d. × 0.25 μm). Chiral-GLC analyses were carried out through a CyclodexB (30 m × 0.25 mm × 0.25 μm) or a Chiraldex G-TA (20 m × 0.25 mm × 0.25 μm) column. EI-MS spectra were recorded at 70 eV by GLC-MS and performed on an Agilent 6890 N gas-chromatograph interfaced with an Agilent 5973 N mass detector. Elemental analyses were acquired with an Elementar Vario Micro Cube in CHNS mode.

^1^H NMR spectra were recorded on a Varian Gemini 200 or a Bruker 400 MHz spectrometer using tetramethylsilane as an internal standard. ^1^H NMR spectral data of the synthetized compounds are reported in Supplementary Materials section.

#### 2.4.2. Enzymatic Resolution of R/S-oct-1-en-3-ol into R-oct-1-en-3-ol- and S-oct-1-en-3-yl-Propionate

Vinyl propionate (11 mL, 10.1 g, 97.5 mmol, 6.5 equiv) and Cal-B (214 mg, 5000 U/g) were added to a solution of *R*/*S*-oct-1-en-3-ol (2.1 g, 16 mmol, 1.0 equiv) in 2-isopropoxypropane (30 mL), according to a reported procedure [36]. This reaction mixture was stirred at room temperature for 21 h, then the volatile components were removed at a reduced pressure. The crude reaction mixture was purified by flash chromatography on silica gel using two different mixtures of petroleum ether (PE) and ethyl acetate (EA) as eluents. From the first chromatographic fractions eluted with a PE–EA 97:3 (*v/v*) mixture, *S*-oct-1-en-3-yl-propionate was recovered (1.32 g, 45% yield) as a colorless liquid. A change in the composition of the eluent to PE–EA mixture 90:10 (*v/v*) allowed the isolation of *R*-oct-1-en-3-ol (0.88 g, 43% yield) as a colorless liquid. Gas liquid chromatography (GLC) analysis of recovered species showed that *S*-oct-1-en-3-yl-propionate (ee = 99%, determined by chiral-GLC analysis) and *R*-oct-1-en-3-ol (ee = 99%, determined by chiral-GLC analysis) had a chemically purity of 98%. The NMR data were in very good agreement with those previously reported [37,38] (see Supplementary Materials).

#### 2.4.3. Preparation of S-oct-1-en-3-ol

A mixture of *S*-oct-1-en-3-yl-propionate (1.3 g, 7.1 mmol, 1 equiv), K_2_CO_3_ (2 g, 14.5 mmol, 2 equiv), methanol (8 mL) and H_2_O (2 mL) was stirred at room temperature for 3 h. Next, diethyl-ether (Et_2_O) (30 mL) and H_2_O (10 mL) were added. The resulting two phases were separated, and the aqueous phase was extracted with Et_2_O (3 × 20 mL). Combined organic extracts were dried over Na_2_SO_4_, filtrated and concentrated at a reduced pressure to obtain *S*-oct-1-en-3-ol as a colorless liquid (0.81 g, 89% yield). GLC analyses showed that *S*-oct-1-en-3-ol (ee = 97%, determined by chiral-GLC analysis) was chemically pure (98%). The NMR data were in very good agreement with those previously reported [39,40] (see Supplementary Materials).

#### 2.4.4. Synthesis of (E)-1,1-Diethoxynon-2-en-4-ol

The Grubbs catalyst (138 mg, 0.16 mmol, 0.025 equiv) and 3,3-diethoxyprop-1-ene (3 mL, 2.6 g, 20 mmol, 3 equiv) were added to a solution of oct-1-en-3-ol (837 mg, 6.5 mmol, 1.0 equiv, either enantiomerically enriched or the racemate) in anhydrous DCM (35 mL), according to a procedure of Grubbs’ olefination already reported [36]. The resulting reaction mixture was stirred at room temperature, and the conversion of the precursors and formation of the product were monitored by GLC analysis every 2 h. After 6 h, the solvent was removed under reduced pressure, at room temperature, and the crude product was purified by flash chromatography on silica gel using an 80:20 mixture of PE–EA (*v/v*) as eluent to yield a pale pink liquid (1 g), which was further purified by distillation under reduced pressure (120 °C at 0.4 mbar) through a Kugelrohr apparatus. (*R*)-, (*S*)- or (*R*/*S*)-(*E*)-1,1-diethoxynon-2-en-4-ol were obtained as a colorless liquids (792 mg, 53% yield). GLC analyses showed that (*R*)-(*E*)-1,1-diethoxynon-2-en-4-ol (ee = 94%, determined by chiral-GLC analysis), (*S*)-(*E*)-1,1-diethoxynon-2-en-4-ol (ee = 95%, determined by chiral-GLC analysis) and (*R/S*)-(*E*)-1,1-diethoxynon-2-en-4-ol were chemically pure (>97%). Spectral data of the above compounds were in very good agreement with those previously reported [41,42]. 

Solutions of (*R*)-, (*S*)- or (*R*/*S*)-(*E*)-1,1-diethoxynon-2-en-4-ol were stored at −80 °C. In order to obtain the corresponding aldehydic compound, (*R*)-, (*S*)- or (*R*/*S*)-(*E*)-1,1-diethoxynon-2-en-4-ol, were maintained on ice under a slow stream of nitrogen in order to remove the solvent, then 1 mM HCl was added. After 30 min, the concentration of the corresponding aldehyde was evaluated by spectrophotometric measurement using an extinction coefficient of 13.75 mM^−1^ cm^−1^ [43].

### 2.5. Preparation of GSHNE Adducts 

Adduction of HNE to GSH was obtained as already described [24]. In particular, racemic HNE, 4*R*-HNE or 4*S*-HNE were incubated with GSH at a molar ratio of 1.5:1 thiol:aldehyde in 50 mM sodium phosphate buffer (pH 7.4). The incubation mixture was kept for 1 h at 37 °C and then left standing overnight at 4 °C. The progress of the reaction was monitored by measuring both the residual free aldehyde at 224 nm and the residual reduced thiol [44]. A stoichiometric consumption of GSH and HNE was observed, with less than 10% of the residual alkenal. As reported [32], the spontaneous formation of the adduct between GSH and HNE leads to the formation of the possible products in comparable amounts. The adducts were stored at –80 °C until their use.

### 2.6. Mass Spectrometry Analysis of GSHNE Reaction Products Obtained after hAKR1B1 Catalysis

Triplicate samples containing 100 µM GSHNE, prepared as described in Section 2.5 using *R,S*-HNE for adduct preparation, and 200 µM NADPH in 50 mM phosphate buffer (pH 6.8) were supplemented with 111 mU/mL of *h*AKR1B1 and incubated at 37 °C. In order to determine the reaction extent on a time-course basis, identical aliquots from the abovementioned mixtures were sampled at different incubation times (i.e., 0, 5, 10, 15, 20, 30, 45, 60, 90 and 120 min, respectively). After sampling, reaction aliquots were immediately frozen in dry ice, lyophilized and stored at −80 °C until further use. In parallel, samples containing isolated components—namely, 100 µM GSHNE, 200 µM NADPH or 111 mU/mL *h*AKR1B1 in 50 mM phosphate buffer (pH 6.8), or their binary mixtures—were incubated at 37 °C and sampled of identical aliquots that were frozen, lyophilized and stored at −80 °C before their use, as reported above. 

All samples mentioned above were dissolved in 0.1% (*v/v*) aqueous trifluoroacetic acid and subjected to nano-LC-ESI-LIT-MS/MS analysis with a LTQ XL mass spectrometer (ThermoFisher, Waltham, MA, USA) mounting a Proxeon nanospray source that was connected to an UltiMate 3000 RSLC nano-liquid chromatographer (ThermoFisher, Waltham, MA, USA) [23,24]. Analyte mixtures were resolved on a 75 μm I.D. × 15 cm column packed with Acclaim PepMap RSLC C18, 2 μm, 100 Å (ThermoFisher, Waltham, MA, USA), using a gradient of solvent B (0.1% *v/v* aqueous formic acid in 80% acetonitrile) in solvent A (0.1% *v/v* aqueous formic acid) at a total flow rate of 300 nL/min. After sample loading, solvent B rose in a linear fashion (3–10% in 7 min; 10–40% in 30 min; 40–80% in 5 min). The range of spectra acquisition was set to *m/z* 300−800, and a data-dependent scanning procedure was used based on the collision-induced dissociation of the three most intense ions, allowing dynamic exclusion (repeat count: 1; exclusion duration: 60 s) and fixing collision energy to 35% and mass isolation window to *m/z* 3.

Assignment of MS/MS spectra to specific glutathione derivatives was carried out by manual interpretation of raw data [23,24]. In order to achieve a semi-quantitative measurement of the reaction products, the same samples as for the MS/MS experiments were also analyzed under the experimental conditions described above, but only in MS scan mode (without ion fragmentation). To this aim, extraction from the same total ion chromatogram of the LC-MS peaks was manually carried out relative to GSHNE and its observed reaction products; subsequent peak integration was gained utilizing the Genesis algorithm, which is part of the Xcalibur software v. 2.0.7 SP1 (ThermoFisher, Waltham, MA, USA). Because of the different ionization properties of the compounds under analysis, the determination of their relative amounts was obtained by referring resultant peak integration to the peptide Met-Arg-Phe-Ala internal standard (exp. MH^+^ value: *m/z* 524.31; theor. MH^+^ value: *m/z* 524.26) that was added at the same concentration to all samples prior to MS analysis. The ratio: peak area (for the species of interest)/peak area (internal reference peptide) was used to evaluate the extent of modification in a semi-quantitative manner. All samples reported above were subjected to this procedure by carrying out experiments in technical quintuplicate.

### 2.7. Molecular Modeling 

The crystal structure of *h*AKR1B1 (pdb code 2F2K) [45] was downloaded from the RCBS Protein Data Bank [46]. After the addition of hydrogen atoms, the structure was minimized using Amber14 software [47] and an ff14SB force field at 300 K. The complex was placed in a rectangular parallelepiped water box, and an explicit solvent model for water (TIP3P) [48] was used with a 20-Å water cap. In order to neutralize the system, sodium ions were added. Two steps of minimization were then carried out. In the first stage, the protein was fixed with a position restraint of 500 kcal/mol Å^2^, thus minimizing only the position of water molecules. In the second stage, the entire system was minimized through 5000 steps of steepest descent followed by conjugate gradient (CG) until a convergence of 0.05 kcal Å^−1^ mol^−1^. The GSHNE ligands were built using Maestro v9.0 (Schrödinger Inc.: Portland, OR, USA) and were minimized by means of Macromodel v9.7 (Schrödinger Inc.: Portland, OR, USA) in a water environment; the CG method was employed until a convergence value of 0.05 kcal Å^−1^ mol^−1^ was reached, using the MMFFs force field [49] and a distance-dependent dielectric constant of 1.0. The binding site region used by the docking program GOLD [50] was defined in order to contain the residues within 10 Å from the ligand in the X-ray structure. The “allow early termination” option was deactivated, while the possibility for the ligand to flip ring corners was activated. The remaining GOLD default parameters were used, and the ligands were submitted to 30 genetic algorithm runs. The docking calculations were carried out using the ChemScore fitness scoring function [24]. A cluster analysis was performed on the results using an RMSD tolerance of 2.0 Å, and the best-ranked pose was considered. The resulting four ligand-protein complexes were subjected to molecular dynamic (MD) simulations as already reported by us [51,52]. 

MD simulations were performed using Amber v. 14 (University of California, San Francisco, CA, USA), and were carried out using the ff14SB force field [53] at 300 K. The complexes were placed in a rectangular parallelepiped water box. An explicit solvent model for water (TIP3P) was used, and the complexes were solvated with a 20-Å water cap. Sodium ions were added as counterions to neutralize the system. Prior to MD simulations, two steps of minimization were carried out using the same procedure described above. Particle mesh Ewald electrostatics and periodic boundary conditions were used in the simulation, as already reported [54,55]. The MD trajectory was run using the minimized structure as the starting conformation, with a time step of 2.0 fs and a cutoff of 10 Å for the nonbonded interaction. SHAKE was used to keep all bonds involving H atoms rigid (the size of the MD timestep is determined by the fastest motions in the system and SHAKE removes the bond stretching freedom—which is the fastest motion—consequently allowing a larger timestep to be used [56]). For the initial 0.5 ns of the MD simulation, constant-volume periodic boundary conditions were used during which the temperature was raised from 0 to 300 K. Then, 10 ns of constant-pressure periodic boundary MD was carried out at 300 K using a Langevin thermostat to maintain a constant temperature in our system. A harmonic force constant of 10 kcal Å^−2^ mol^−1^ was used for blocking all α-carbons of the protein. General Amber force field parameters were assigned to the ligands and co-factor, while partial charges were calculated using the AM1-BCC method [57], as implemented in the Antechamber suite of Amber v.14. The final structures of the complexes were obtained as the averages of the last 10.0 ns of MD minimized by the CG method until reaching a convergence of 0.05 kcal Å^−2^ mol^−1^. The average structures were obtained using the Cpptraj program [58] implemented in Amber v.14.

### 2.8. Density Functional Theory (DFT) Calculations

DFT calculation was performed using the Terachem package v.1.9, PetaChem, Los Altos Hills, CA, USA [59,60] at the B3LYP/6-311+G(d,p) level. Empirical dispersion corrections were included [61]. The calculation was performed on a GPU Workstation with 4 G-Force Titan GTX. 

DFT calculation was performed via the Terachem package v. 1.9 [59,60]. The Terachem package performs DFT calculations using Graphical Processing Units. The B3LYP/6-311+G(d,p) level of calculations was used along with Grimme D3 empirical dispersion corrections [61]. A GPU-based workstation with 4 G-Force Titan GTX GPUs connected by a SLI interface was used.

### 2.9. Other Methods

Protein concentration was determined according to Bradford [62], using BSA as standard protein. Kinetic parameters for *h*AKR1B1 were obtained via nonlinear regression analysis of the rate measurements performed at different substrate concentrations (GraphPad Instat version 6.0, San Diego, CA, USA). 

Statistical analysis was performed using a two-way ANOVA test carried out with Graphpad 6.0 software.

## 3. Results and Discussion

Stereoselective synthesis of the two HNE enantiomers (see Section 2.4 for synthesis details) allowed suitable chiral substrates to be assayed for their *h*AKR1B1-catalyzed reduction. The obtained results indicated that the configuration of the stereochemical center present at C4 in HNE slightly affected recognition of the aldehyde by *h*AKR1B1. In fact, the *k_cat_* values measured for the two isomers were essentially comparable, and a two-fold increase of K_M_ was observed for *4R*-HNE with respect to the *S*-enantiomer (Table 1). 

**Figure 2:**
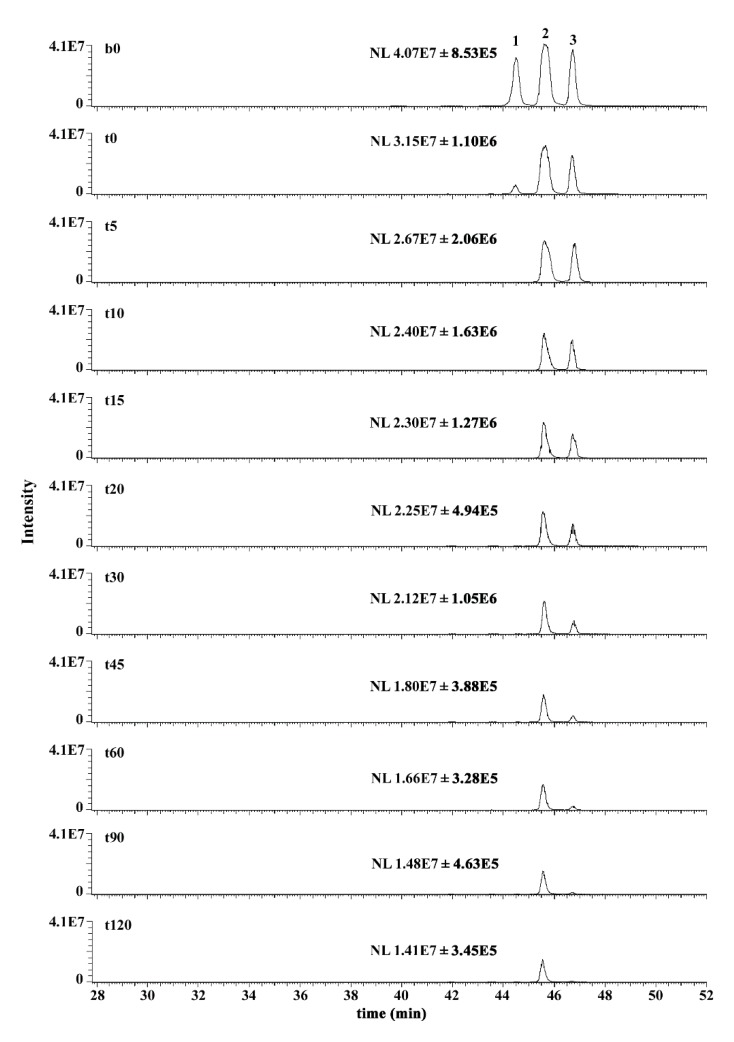
Nano-LC-ESI-LIT-MS analysis of the reaction mixture containing *h*AKR1B1, GSHNE and NADPH. Reported are the extracted ion currents (EIC) relative to GSHNE (i.e., *m/z* 464.25), which were measured for the 10 sampling times (from t0 to t120) of the reaction mixture (see Section 2.6) and for the reference reaction mixture b0 without *h*AKR1B1. Sample t0 refers to a sample withdrawn from the reaction mixture just after the addition of *h*AKR1B1. For each sampling time, normalized intensity levels (NLs) are reported as the mean values ± SD determined over five chromatographic runs; NL refers to the largest peak of the GSHNE triplets 1, 2 and 3.

**Figure 3:**
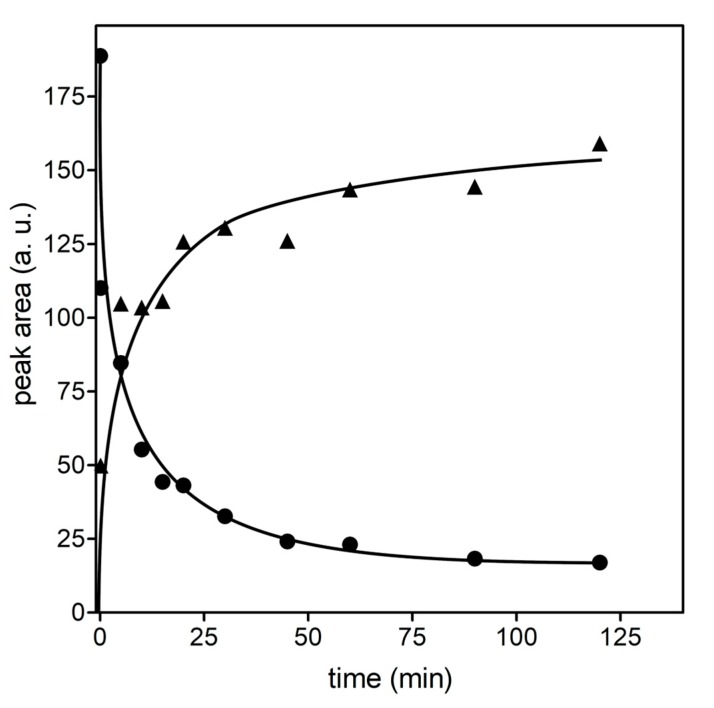
Time-course disappearance of the substrate and appearance of the product during GSHNE transformation catalyzed by *h*AKR1B1. Time-course of GSHNE disappearance (circles) and GSDHN formation (triangles) as obtained by plotting the different sampling times for the peak areas of the molecular species at *m/z* values of 464.25 and 466.22, respectively. Peak areas are the mean values ± SD over five chromatographic runs for each sampling time; error bars, when not visible, are within the symbol’s size.

The case for the glutahionyl-HNE adducts was different. As mentioned above, the reaction of HNE with GSH leads to the generation of a molecule in which a novel stereocenter (C3) is present. This should imply the generation of four possible diastereoisomers (Figure 1). However, due to the presence of an alcoholic moiety in the γ position with respect to the aldehydic group, each diastereoisomer exists as a couple of cyclic anomers and an open aldehydic form. It is worth noting that *h*AKR1B1 acts on an open aldehydic form [63], and its inability to catalyze the ring opening in the case of glucose hemiacetal has been demonstrated [64]. Solutions of GSHNE obtained from *R,S*-HNE were demonstrated by ours and other groups to be resolved by LC-ESI-MS/MS into four peaks referring to each of the four diastereoisomers and containing the open forms and corresponding coupled cyclic anomers [23,24,32,33]. We verified this condition also in this study, when the products resulting from the reaction of *R/S*-HNE with GSH were resolved on a C_18_-based column. In particular, two of the four anomeric couples were well resolved, namely 3*S,*4*R*-GSHNE and 3*R,*4*R*-GSHNE (peak 1 and peak 3, respectively, based on assignments reported in abovementioned studies, see Figure S1 and Figure 2, b0 trace). Conversely, 3*S,*4*S*-GSHNE and 3*R,*4*S*-GSHNE were practically co-eluting in peak 2 (Figure 2) and were appreciated as distinct chromatographic species (peak 2a and 2b, respectively; Figure S1) only when a very limited amount of material was analyzed. Taking advantage of this analytical procedure, we investigated the stereochemistry of *h*AKR1B1-catalyzed reduction of racemic GSHNE (Figure 2). The results indicate that 3*S,*4*R*-GSHNE underwent the most rapid transformation, as its peak appeared significantly reduced immediately after the addition of enzyme (Figure 2, t0 trace) and became completely absent in the chromatogram after 5 min of incubation. Conversely, 3*S,*4*S*-GSHNE and 3*R,*4*S*-GSHNE (peak 2) resulted the most resistance to transformation. However, an almost-complete transformation of the substrate occurred as the result of a long duration of incubation (i.e., 120 min, Figure 3). This observation is consistent with the possibility of a titration of GSHNE solutions by means of spectrophotometric measurements in the presence of aldose reductase [43]. For this reason, a kinetic analysis was performed using the glutathionyl adducts obtained starting either from *4R*-HNE or *4S*-HNE as substrates of *h*AKR1B1. Results reported in Table 1 reveal a more marked difference in kinetic parameters among 4*R*- or 4*S*-HNE-derived glutathione adducts with respect to 4*R*-HNE or 4*S*-HNE precursors (Table 1). In fact, 3*R/S,*4*S*-GSHNE clearly resulted in a worse substrate in terms of both *K_M_* (more than 10-fold higher) and *k_cat_* (1.7-fold lower) values in comparison with the 3*R/S,*4*R*-GSHNE. Thus, an almost 20-fold higher specificity constant value was observed for the 4*R*-HNE-derived glutathione adducts in comparison with the 4*S*-HNE-derived counterparts. These results confirm the chromatographic profiles shown in Figure 2, where a preferential stereochemical transformation of GSHNE diastereoisomers was observed. 

In order to find a molecular rationale for the stereochemical preference of *h*AKR1B1 described above, possible differences in the structural stability of the open form (the one recognized as substrate) were analyzed for all four GSHNE diastereoisomers with respect to their hemiacetalic counterparts.

The different relative energies of the three possible forms (i.e., two cyclic anomers and an open species) of each diastereoisomer were correlated to the populations of these molecules. These energies were calculated using the Terachem package [59,60,61] at the B3LYP/6-311++G(d,p) level of the density functional theory. All the molecular geometries were fully optimized and characterized as minimal ones. The relative energies with respect to the most stable form chosen as zero (i.e., 3*R*,4*R*-GSHNE) are reported in Table 2. As expected [11], it was evident that all the hemiacetalic forms of GSHNE stereoisomers were always calculated as more stable than the open counterparts. In addition, the absolute configuration of the anomeric carbon was calculated relevant to the energy of the molecule only in the case of 3*R,*4*S*-GSHNE. Assuming that all the different forms were in equilibrium, the corresponding relative populations were calculated according to the Boltzmann law. Almost all populations of molecules resided in at least one of the two closed forms. However, the population of the open structure, which is the one relevant for the *h*AKR1B1 activity, had significantly different results among the various diastereoisomers, despite being very small. When we considered the ratio of open structure populations with respect to the less stable one (i.e., 3*S,*4*S*-GSHNE), we found that 3*S,*4*R*-GSHNE seemed to be the most probable one, followed by 3*R,*4*S*-GSHNE.

**Figure 4:**
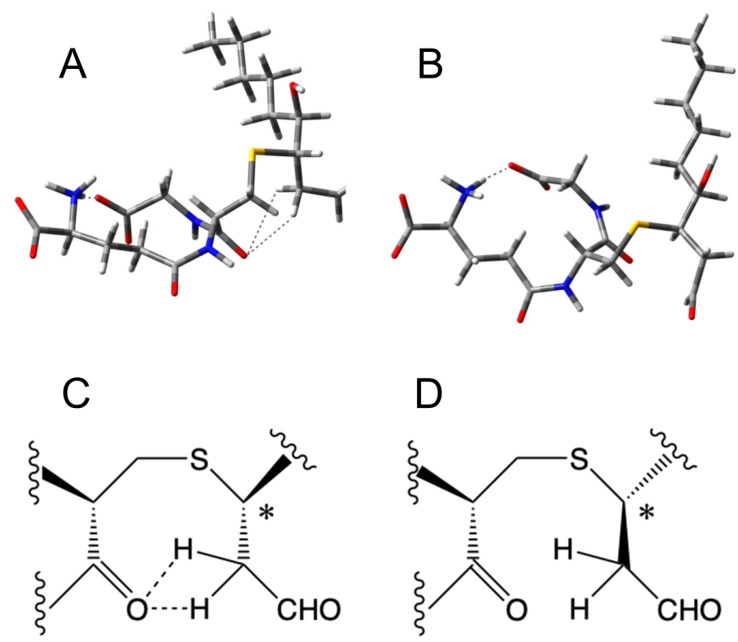
Structure of the open forms of GSHNE diastereoisomers. (**A**) The structure of 3*S*,4*R-GSHNE*. The interactions between glutathione amino- and carboxyl-terminals and between the C2 acidic protons of the nonanal portion and the one amidic group of the glutathione moiety are shown. (**B**) The structure of 3*R*,4*R*-GSHNE. The interaction between glutathione amino- and carboxyl-terminals is shown. (**C,D**) The different configurations of the chiral center (*) allow intramolecular hydrogen bonding when the involved groups are on the same side of the plane for 3*S*,4*R-GSHNE* (**C**), and prohibit bonding when the involved groups are on opposite sides of the plane for 3*R*,4*R*-GSHNE (**D**). Only relevant parts of the molecules are shown in the last two panels.

A careful inspection of the open GSHNE structures showed that significant interactions between the acidic protons of the C2 acidic protons of the nonanal moieties and the amidic groups of the glutathionyl portion were observed in the cases of 3*S,*4*R*-GSHNE (Figure 4A,C) and 3*R,*4*S*-GSHNE (data not shown). The stereochemistry did not allow this kind of interaction in the case of both 3*R,*4*R*-GSHNE (Figure 4B,D) and 3*S,*4*S*-GSHNE (data not shown). In all closed structures, this interaction was not possible for the different spatial arrangements of diastereoisomers and for the very low acidities of the protons mentioned above when the adjacent carbon was hemiacetalic. 

**Figure 5:**
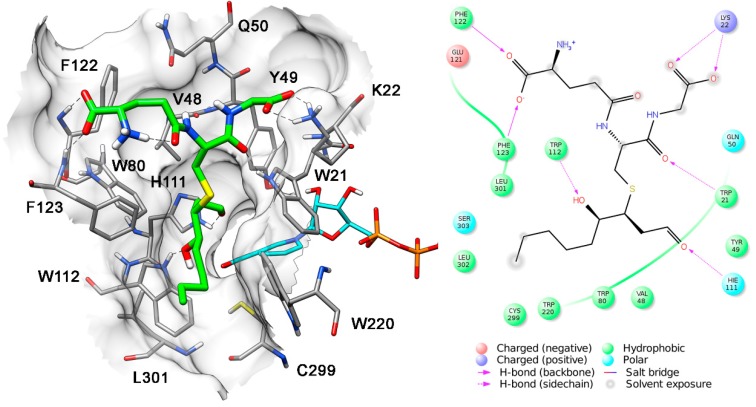
Interaction of *h*AR1B1 and *3S,4R-*GSHNE. The minimized average structure of 3*S,*4*R-*GSHNE docked into the *h*AKR1B1 active site is reported. All interactions between the GSHNE and enzyme residues are shown.

In order to identify the potential binding mode of the open structures of different GSHNE diastereoisomers, their possible interaction with *h*AKR1B1 was evaluated by means of docking and MD simulations. Figure 5 shows the results obtained for 3*S*,4*R*-GSHNE. In particular, this diastereoisomer showed a T-shaped disposition, with its glutathione moiety perpendicular to the corresponding hydroxynonanal portion. The glutathionyl mojety showed H-bonds with the nitrogen of the polypeptide bond linking F122 and F123, as well as ionic interactions with K22, whereas the alkyl chain of the nonanal portion was inserted into a lipophilic pocket delimited by W112, F123, W220 and L301. The 4-hydroxyl group of 3*S*,4*R*-GSHNE showed a stable H-bond with the indole ring of W112, and the aldehydic portion of the adduct was inserted into the catalytic region of the *h*AKR1B1 binding site where it was also hydrogen-bonded to H111.

**Figure 6:**
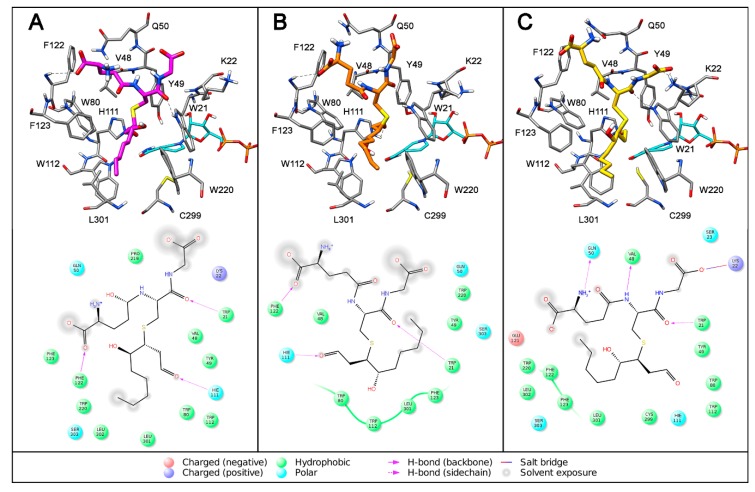
Interaction of *h*AR1B1 with different GSHNE diastereoisomers. The minimized average structures of 3*R,*4*R*-GSHNE (**A**), 3*R*,4*S*-GSHNE (**B**) and 3*S*,4*S*-GSHNE (**C**) docked into the *h*AKR1B1 active site are reported. All interactions between the GSHNE and enzyme residues are shown.

As shown in Figure 6, the other three diastereoisomers showed a binding orientation very similar to that observed for 3*S*,4*R*-GSHNE, characterized by the same T-shaped conformations and similar dispositions of the glutathione and hydroxynonanal portions. 

In all protein complexes with these three diastereoisomers, the aldehydic portion protruded inside the enzyme’s catalytic region and the alkyl chain of the nonanal group was inserted into the lipophilic pocket delimited by W112, F123, W220 and L301. However, unlike 3*S*,4*R*-GSHNE, the 4-hydroxyl group of the other three diastereoisomers did not appear to be important to the binding of *h*AKR1B1, as it did not show any interaction with the protein. The presence of a stable H-bond between the 4-hydroxyl group of 3*S*,4*R*-GSHNE and the indole ring of W112, while stabilizing the enzyme-substrate complex, can favor hemiacetal openings for this diastereoisomer, thus promoting the incidence of this molecule as the preferential substrate for hAKR1B1, as experimentally verified.

The ability of AKR1B1 to reduce GSHNE into GSDHN results in the pro-inflammatory role of the enzyme. In fact, GSDHN is considered to be an activator of the NF-kB inflammatory response, which is mediated by the action of phospholipase C/protein kinase C (PLC/PKC) enzyme couple. Despite several indirect studies on this connection [65,66,67], the activation effect exerted by GSDHN on the PLC/PKC system has not yet been clarified from a molecular point of view. Furthermore, no experimental indications have been provided on the possible differences of the effects exerted by the various GSDHN diastereoisomers so far. Thus, the preferential activity of AKR1B1 on the 3*S*,4*R*-GSHNE diastereoisomer reported herein may be stimulating for further and more-specific studies on cell responsiveness to the AKR1B1-mediated transformation of GSHNE. Recently, we reported a selectivity for CBR1 in the NADP^+^-dependent oxidative transformation of GSHNE diastereoisomers to their corresponding γ-lactones [24]. In that case, we observed that 3*S*,4*R*-GSHNE (i.e., peak 1 in both Figure 2 and Figure S1) was transformed at a markedly reduced rate in respect to what occurred for 3*S*,4*S*-GSHNE, 3*R*,4*S*-GSHNE and 3*R*,4*R*-GSHNE (peaks 2 and 3 in both Figure 2 and Figure S1). Thus, the different stereoselectivity of CBR1 and AKR1B1 active sites gives these two enzymes the ability to differently recognize the various GSHNE diastereoisomers. In this regard, AKR1B1 and CBR1 appear to somehow be able to contribute in a complementary manner to the removal of all GSHNE diastereoisomers whose functions as precursors of a signaling molecule may be modulated.

## 4. Conclusions

The ability of AKR1B1 to preferentially reduce, among the four possible diastereoisomers derived from HNE glutathionylation, the 3*S*,4*R*-GSHNE opens the way to a more specific investigation on the role of GSDHN as signalling pro-inflammatory molecule. The complementarity in the stereochemical selectivity of AKR1B1 and CBR1 toward GSHNE suggests the possibility of a concerted action of these enzymes in the removal of the adduct, thus offering, through the stereochemical selection of the substrates, a refined kinetic control of the level of this multi faced molecule.

## Figures and Tables

**Table 1:** Kinetic parameters of *h*AKR1B1 for HNE and GSHNE stereoisomers.

Substrate	*K_M_* (µM)	*k_cat_* (min^−1^)	*k_cat_*/*K_M_* (µM^−1^ min^−1^)
4*R*-HNE	98.2 ± 6.7	87.5 ± 6.2	0.9 ± 0.1
4*S*-HNE	47.6 ±5.7	91.3 ± 8.0	1.9 ± 0.3
3*R,S,*4*R*-GSHNE	28.3 ± 4.6	120.9 ± 6.3	4.3 ± 0.7
3*R,S,*4*S*-GSHNE	319.6 ± 55.7	70.0 ± 9.0	0.2 ± 0.1

**Table 2:** Relative energies and Boltzmann populations of GSHNE diastereoisomers.

GSHNE Diasteroisomer	Relative Energies (KJ/mol)			Boltzmann Population (300 K)			*R*
	Open	Close (R)	Close (S)	Open	Close (R)	Close (S)	
3R,4R	49.62	6.66	0.00	2.12 ∙10^−9^	0.06	0.94	139.77
3S,4S	65.68	4.71	6.09	1.52 ∙10^−11^	0.63	0.37	1.00
3S,4R	54.02	18.11	18.28	2.87 ∙10^−7^	0.52	0.48	18,932.25
3R,4S	54.04	34.21	15.08	1.63 ∙10^−7^	4.65 ∙10^−4^	1.00	10,744.65

## Supplementary Materials

The following are available online at https://www.mdpi.com/2076-3921/8/10/502/s1. NMR spectra data; Figure S1: Stereochemical configuration of the GSHNE diastereoisomers (**A**) and their nano-LC-ESI-LIT-MS analysis (**B**). Reported are the corresponding extracted ion currents (EICs) relative to GSHNE (i.e., *m/z* 464.25) and identified chromatographic peaks.
