# Supplementary material for: Stereoselectivity of Aldose Reductase in the Reduction of Glutathionyl-Hydroxynonanal Adduct
Source: Antioxidants (Basel). 2019 Oct 22;8(10):502. doi: 10.3390/antiox8100502 (PMC6827081; doi:10.3390/antiox8100502)
Supplement: Supplementary file 1 [file antioxidants-08-00502-s001.pdf]

Supplementary Materials

NMR Spectra

The following notation was used in order to report NMR spectra: s = singlet, bs = broad singlet, d = doublet, dd = double doublet, t = triplet, dt = double triplet, q = quadruplet. The ^{13}C NMR spectra were recorder at 50 or 100 MHz, using Varian Gemini or Bruker instruments, respectively, and the spectra referred to the signal of the solvent.

S-oct-1-en-3-yl propionate

^{1}H-NMR (400 MHz, $CDCl_3$): δ = 0.88 (m, 3H), 1.19 (t, *J* = 8 MHz, 3H), 1.26-1.63 (m, 8H), 2.32 (q, *J* = 8 MHz, 2H), 5.11-5.29 (m, 3H), 5.70-5.87 (m, 1H).

EI-MS: *m/z* (%) 128.2 (19), 113.1 (22), 99.1 (11), 57.1 (100), 54.2 (17).

Ee = 99% (CyclodexB - 30 m × 0.25 mm × 0.25 μm) R.t (*R*)= 23.81 min; R.t (*S*) = 24.73 min.

$[\alpha]_D^{22.5} = -10.45$ $CHCl_3$ (c = 11.7 mg/mL).

R-oct-1-en-3-ol

^{1}H-NMR (400 MHz, $CDCl_3$): δ = 0.86 (m, 3H, CH_3CH_2), 1.26-1.47 (m, 8H), 3.98 (m, 1H), 5.07–5.30 (m, 2H), 5.79-5.96 (m, 1H).

EI-MS: *m/z* (%) 83.9 (28), 72.1 (13), 57.1 (100), 49.0 (11), 43.1 (14).

Ee = 99% (CyclodexB - 30 m × 0.25 mm × 0.25 μm). R.t (*R*)= 22.91 min; R.t (*S*)= 25.57 min.

$[\alpha]_D^{22.5} = -11.6$ $CHCl_3$ (c = 12.5 mg/mL) ($[\alpha]_D^{22.5} = -11.7$) [1]

S- oct-1-en-3-ol

^{1}H-NMR (400 MHz, $CDCl_3$): δ = 0.86 (m, 3H), 1.26–1.47 (m, 8H), 3.98 (m, 1H), 5.07–5.30 (m, 2H), 5.79–5.96 (m, 1H).

EI-MS: *m/z* (%) 83.9 (28), 72.1 (13), 57.1 (100), 49.0 (11), 43.1 (14).

Ee = 97% (CyclodexB - 30 m × 0.25 mm × 0.25 μm). R.t (*R*)= 22.91 min; R.t (*S*)= 25.57min.

$[\alpha]_D^{22.5} = +9.1$ $CHCl_3$ (c = 5 mg/mL) ($[\alpha]_D^{25} = +9$) [2,3]

E-1,1-diethoxynon-2-en-4-ol

^{1}H-NMR (400 MHz, $CDCl_3$) δ: 0.87–0.90 (m, 3H, CH_3), 1.20 (t, 6H, *J* = 14 MHz, 2 x CH_3), 1.30–1.34 (m, 6H, 3 x CH_2), 1.55 (m, 2H, CH_2), 3.48–3.52 (m, 2H, CH_2), 3.62–3.66 (m, 2H, CH_2), 4.15 (m, 1H, CH), 4.89–4.91 (m, 1H, CH), 5.66–5.71 (m, 1H, CH), 5.84–5.88 (m, 1H, CH).

^{13}C-NMR (100 MHz, $CDCl_3$): 13.9, 15.1, 22.5, 24.9, 25.0, 31.6, 31.7, 36.4, 37.0, 60.9, 60.9, 71.8, 1001.0, 127.4, 137.1.

Elemental analysis for $C_{13}H_{26}O_3$: Calculated C, 67.79; H, 11.38; O, 20.84; Found C, 67.82; H, 11.34; O, 20.82

R-E-1,1-diethoxynon-2-en-4-ol

Ee = 94% (Chiraldex G-TA: 20 m x 0.25 mm x 0.25 μm). R.t (*R*) = 16.17 min; R.t (*S*)= 16.39 min.

$[\alpha]_D^{22.5} = -9.17$ $CHCl_3$ (c = 0.12 mg/mL)

S-E-1,1-diethoxynon-2-en-4-ol

Ee = 95% (Chiraldex G-TA - 20 m x 0.25 mm x 0.25 μm). R.t (*R*)= 16.17 min; R.t. (*S*)= 16.39 min.

$[\alpha]_D^{22.5} = +9.3$ $CHCl_3$ (c = 0.12 mg/ mL)

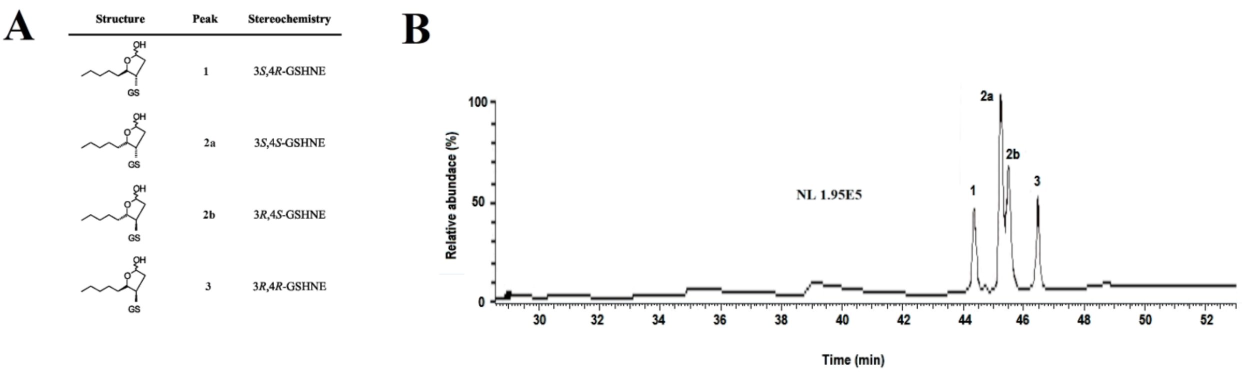

Figure S1. Stereochemical configuration of the GSHNE diastereoisomers (**A**) and their nano-LC-ESI-LIT-MS analysis (**B**). Reported are the corresponding extracted ion currents (EICs) relative to GSHNE (i.e., *m/z* 464.25) and identified chromatographic peaks.

References

[1] Badenhop, A.F.; Wilkens, W.F. The formation of 1-octen-3-o1 in soybeans during soaking. *J. Oil Chem. Soc.* **1969**, 46, 179-182. (ref. [33] of the main text)

[2] Icikawa, A.; Ono, H. Preparation of single-enantiomer semiochemicals using 2-methoxy-2-(1-naphthyl) propionic acid and 2-methoxy-2-(9-phenanthryl) propionic acid. *Tetrah. Asymm*. **2005**, 16, 2559-2568. (ref. [35] of the main text) (

[3] Kanbayashi, N.; Onitsuka, K. Ruthenium-catalyzed regio- and enantioselective allylic substitution with water: direct synthesis of chiral allylic alcohols. *Angew. Chem. Int. Ed.* **2011**, 50, 5197-5199. (ref. [36] of the main text)
